# A Two-Front Battle: A Case Report of Pulmonary Tuberculosis and Concurrent Peripheral Neuropathy

**DOI:** 10.7759/cureus.98778

**Published:** 2025-12-08

**Authors:** Hazim Mahmoud, Malaz Khalifa, Ahmed Ahmed, May Jalal, Safwan Ahmed

**Affiliations:** 1 Internal Medicine, Hamad Medical Corporation, Khartoum, SDN; 2 Pathology and Laboratory Medicine, Hamad Medical Corporation, Khartoum, SDN; 3 Anesthesia, Hamad Medical Corporation, Khartoum, SDN; 4 Radiology, Hamad Medical Corporation, Khartoum, SDN; 5 Neurology, Hamad Medical Corporation, Doha, QAT

**Keywords:** axonal degeneration, immune-mediated neuropathy, nerve biopsy, para infectious, peripheral neuropathy, pulmonary tuberculosis

## Abstract

Peripheral neuropathy is often observed in patients with tuberculosis (TB), usually as a side effect of treatment rather than a direct result of the infection. Neuropathy presenting prior to the initiation of anti-TB therapy is considered uncommon. We describe a 27-year-old patient who presented with persistent, deep burning, severe pain in the bilateral lower limbs against a background of chronic cough, weight loss, and fever. The patient was evaluated for the clinical symptomology and was found to have pulmonary TB based on sputum analysis. Concurrently, evaluation of lower limb burning paresthesias revealed evidence of neuropathy confirmed both electrophysiologically and by tissue biopsy. Evaluations for causes of acute neuropathy were negative, leading to a diagnosis of para-infectious neuropathy related to TB. Neuropathy in TB is most often drug-induced, especially with medications such as isoniazid or linezolid in drug-resistant cases. In this patient, however, symptoms developed before treatment, pointing to a para-infectious mechanism, likely immune-mediated inflammation affecting the peripheral nerves. This case shows a rare but important manifestation of para-infectious TB neuropathy presenting before therapy begins. Recognizing this distinction is crucial to avoid misdiagnosis and ensure timely, appropriate management.

## Introduction

Peripheral neuropathy (PN) is a clinical condition characterized by dysfunction of peripheral nerves distal to the nerve roots. It can involve a single nerve (mononeuropathy) or multiple nerves (mononeuropathy multiplex or polyneuropathy). The most common form is a symmetrical, length-dependent polyneuropathy, typically starting in the distal lower limbs [1]. The most common etiologies include diabetes; nutritional deficiencies such as vitamins B1, B6, and B12, as well as copper and vitamin E; toxin exposures such as lead and mercury; and immune-mediated neuropathies [2]. Clinical manifestations will depend on whether there is a predominant motor or sensory affliction. In the context of tuberculosis (TB), neuropathy is commonly seen as a consequence of drug toxicity, most commonly isoniazid [3].

According to a large review, the reported incidence of PN in patients with drug-susceptible TB (DS-TB) ranges from 0% to 10% [4]. In a cohort study from Swaziland involving 250 patients with DS-TB from a single hospital, the overall prevalence was 12%. Rates were higher in males (18%) compared to females (7%) and were most pronounced in adults over 45 years of age, reaching 20% [4]. In patients with drug-resistant TB (DR-TB), the prevalence of PN is significantly higher, with reported figures ranging from 13% to 17% [4]. This increased risk is likely related to the use of more neurotoxic medications and the longer treatment duration required for DR-TB. Notably, a recent study found that up to 25% of patients already exhibited signs of PN before starting DR-TB therapy [4].

TB is a serious infectious disease that can affect multiple organs. However, it rarely presents with primary involvement of the peripheral nerves [5]. We report the case of a young male patient with a positive acid-fast bacillus (AFB) smear who presented with bilateral lower leg pain and severe burning paresthesia of the feet before the initiation of antitubercular therapy. This case highlights an uncommon presentation of TB and outlines our clinical approach to its evaluation and management.

## Case presentation

A 27-year-old male patient with no prior chronic medical conditions presented with a four-week history of productive cough with yellow sputum, occasional hemoptysis, intermittent fever, generalized fatigue, joint pain, and unintentional weight loss of approximately 7 kg over one month, along with severe burning paresthesia of the feet of two weeks’ duration. He denied regular medication use, significant family history, or occupational exposure to known risk factors.

**Figure 1:**
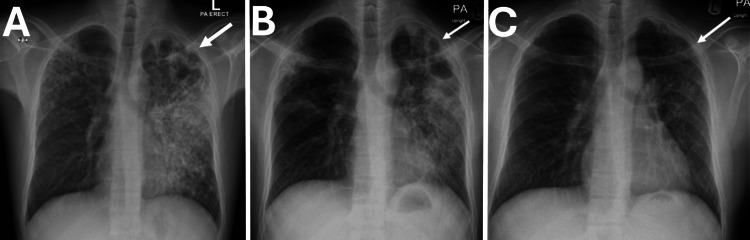
Chest X-ray showing TB treatment progression. (A) before anti-TB treatment; bilateral diffuse patchy, and nodular infiltrates more in the left lung with apical cavitations. Tuberculosis should be considered. (B) After discharge; regression of infiltrates two-months after anti-TB treatment. (C) Six‑month follow‑up; near complete resolution of lesions

Initial chest radiography revealed bilateral patchy consolidations and nodular infiltrates, more pronounced on the left, with cavitation in the upper lobe of the left lung and associated pleural thickening (Figure 1A). These findings were suggestive of pulmonary TB. Microbiological confirmation was obtained with a positive AFB smear with 5 AFB per 100 fields and positive *Mycobacterium tuberculosis* (MTB) DNA.

About 10 days prior to admission, he developed severe burning pain and paresthesias involving both feet below the ankle. The pain persisted, markedly limiting ambulation, and was only partially relieved by regular paracetamol and occasional ibuprofen. On admission, neurological examination showed preserved lower limb motor strength, present ankle reflexes, and marked hyperesthesia to light touch over the soles and dorsal aspects up to the lower third of the leg. Over the following few weeks, ankle reflexes became sluggish to absent, although motor power remained intact. Pain continued to significantly interfere with walking.

Nerve conduction studies demonstrated severe bilateral sensorimotor axonal neuropathy affecting the lower limbs. Laboratory evaluation (Table 1) included serial complete blood counts, in which hemoglobin (g/dL) was initially 10.5 and increased to 11.3 at discharge, renal and liver function tests monitored every 72 hours, and serum vitamin B12, folate, and vitamin D levels (22 ng/mL initially, improving to 35 ng/mL), all within normal ranges. CRP was initially 75.5 mg/L and decreased to 28 mg/L on follow-up. Serum calcium was elevated with suppressed parathyroid hormone. HIV testing was negative, and molecular TB testing showed no rifampicin resistance. Extensive workup for autoimmune etiologies was negative (Table 2).

**Table 1:** Patient laboratory evaluation MCV: mean corpuscular volume; CRP: C-reactive protein; TSH: thyroid-stimulating hormone.

Variable	Patient value	References
Hemoglobin (g/dL)	10.5	13-17 (male)
MCV (fL)	77.7	80-100
Platelets (x10^9^/L)	537	150-400
CRP (mg/L)	75.5	<5
Albumin (g/L)	25	35-50
TSH (µIU/mL)	2.3	0.4-4.0
Parathyroid hormone (ng/L)	2	10-65
Adjusted calcium (mmol/L)	2.99	2.1-2.6
Sodium (mmol/L)	131	135-145
Magnesium (mmol/L)	0.62	0.7-1.1
folate (ng/mL)	10	>4
Vitamin D (ng/mL)	22	20-50
Vitamin B1 (nmol/L)	172	66-260
Vitamin B6 (pmol/L)	168	35-110
Vitamin B12 (nmol/L)	378	133-675

**Table 2:** Autoimmune and infectious serology ANA: antinuclear antibody; ANCA: antineutrophil cytoplasmic antibody; PR3: proteinase 3; MPO: myeloperoxidase; dsDNA: double-stranded DNA; CCP: cyclic citrullinated peptide; HIV: human immunodeficiency virus.

Serology	Result
ANA	Negative
c-ANCA	Negative
p-ANCA	Negative
Anti-PR3	Negative
Anti-MPO	Negative
Anti-dsDNA	Negative
Anti-CCP	Negative
HIV Ag/Ab combo	Non reactive
Hepatitis B core Ab	Non reactive
Hepatitis B surface Ab	Non reactive
Hepatitis C Ab	Non reactive

**Figure 2:**
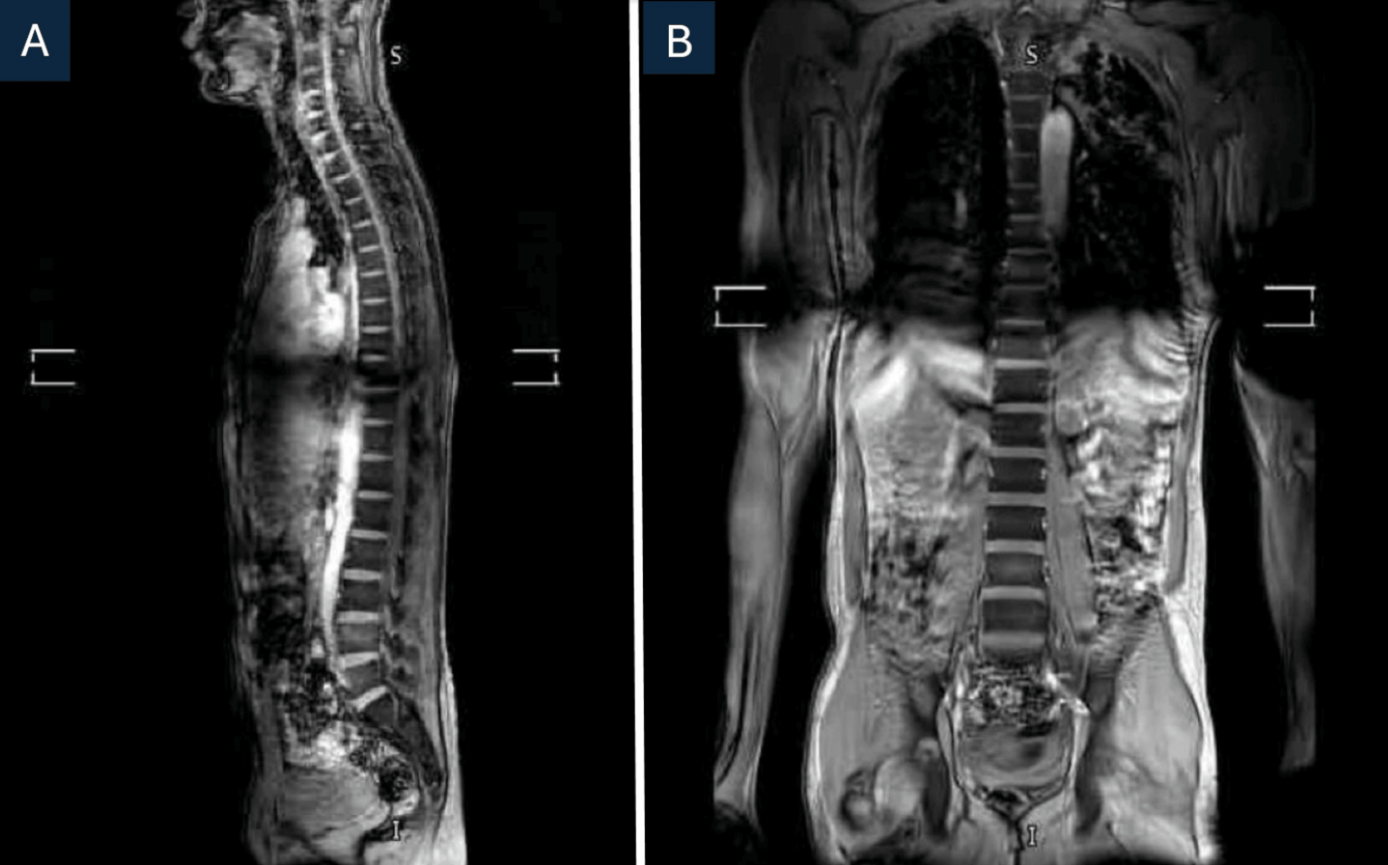
MRI thoracolumbar spine with contrast showing no evidence of spinal infection or cord involvement. (A) Sagittal T1-weighted image demonstrating normal vertebral alignment and spinal cord signal. (B) Coronal T1-weighted image showing intact vertebral bodies and paraspinal soft tissues without abscess or inflammatory changes.

**Figure 3:**
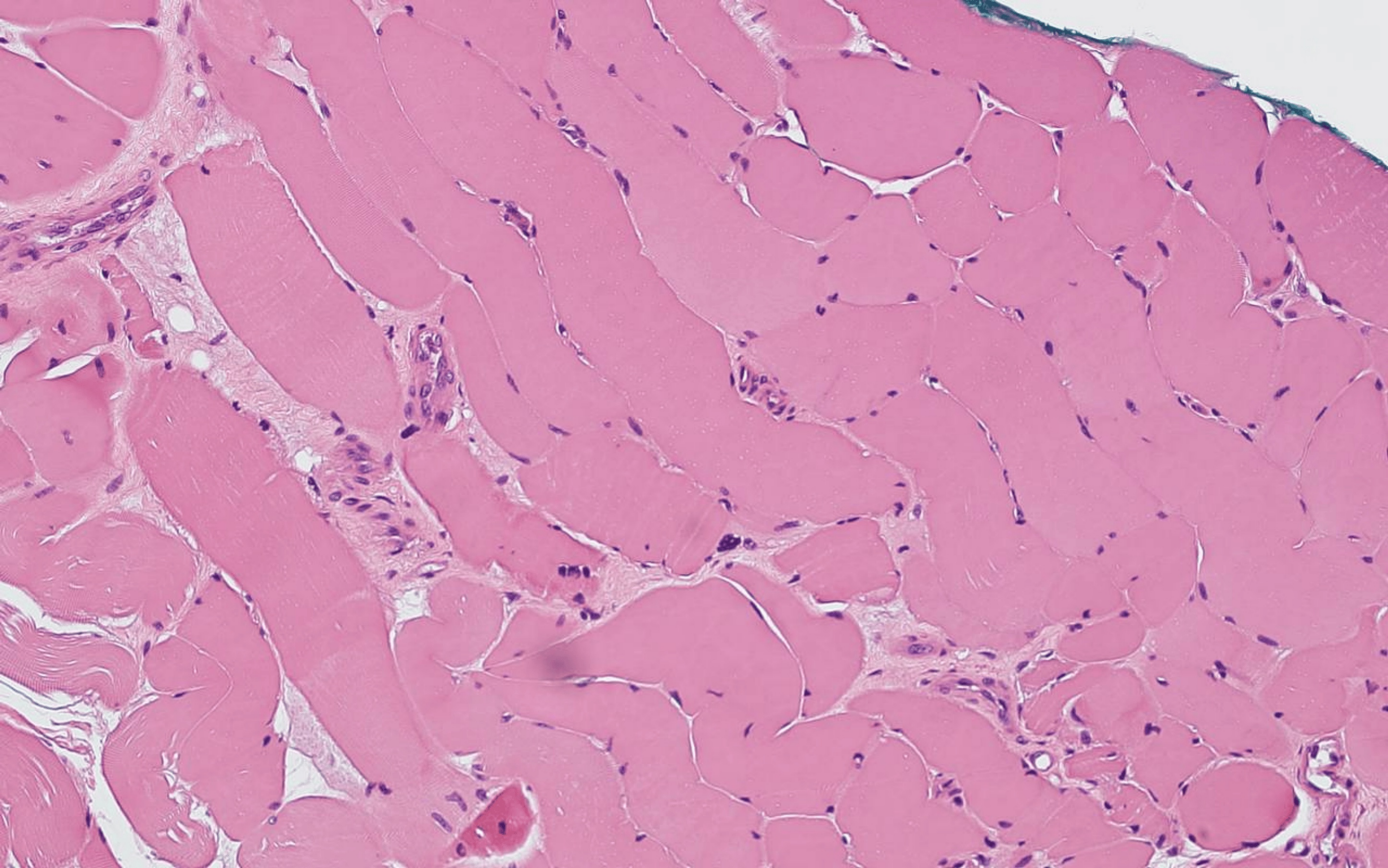
Skin, muscle, and superficial peroneal nerve biopsy revealed few individual epineurial perivascular collections of inflammatory cells are present adjacent to small vessels.

A thoracolumbar MRI was performed to exclude spinal involvement such as Pott’s disease and showed no evidence of infection or cord involvement (Figure 2). There was a possibility of vasculitic or inflammatory neuropathy considering his symptomatology. Skin, muscle, and superficial peroneal nerve biopsy revealed reinnervated skeletal muscle with denervation atrophy, occasional small perivascular inflammatory cell collections in the nerve epineurium, and active axonal degeneration (Figure 3).

Standard first-line anti-tubercular therapy (ATT) consisting of isoniazid, rifampicin, pyrazinamide, and ethambutol was initiated. As he had extensive pulmonary involvement and was improving with ATT and neuropathic agents, steroids were not considered, as the risks outweighed the benefits.

After two months of therapy, follow-up chest radiography showed regression of bilateral patchy and nodular infiltrates, with persistent cavitation and pleural thickening at the left lung apex (Figure 1B). In view of clinical and radiological improvement, the regimen was shifted to the maintenance phase with a fixed-dose rifampicin-isoniazid combination (Rifinah) and pyridoxine supplementation. The patient also received loading doses of injectable thiamine (vitamin B1), pyridoxine (vitamin B6), and cobalamin (vitamin B12).

Neuropathic pain was managed with optimal doses of amitriptyline, gabapentin, and topical lidocaine gel, providing partial relief. Despite ongoing therapy, sputum smears remained positive after one month. The patient continues multidisciplinary follow-up with infectious diseases, neurology, and physiotherapy teams for ongoing management of pulmonary TB and PN.

At six months of follow-up, the patient demonstrated good neurological improvement, with partial sensory regression and improved gait. By that time, he had returned to work, although ankle reflexes remained absent and hyperesthesia persisted below both ankles. A follow-up nerve conduction study will be scheduled by the end of anti-TB treatment to reevaluate recovery. Chest radiography also revealed a marked reduction in lung infiltrates after the intensive phase; however, some apical changes could still be noted (Figure 1C), and the sputum smear was negative during the intensive phase.

## Discussion

PN is a common neurological condition that arises as a side effect of antitubercular medication during TB treatment. The neurotoxic effects of linezolid and other second-line agents used in multidrug-resistant (MDR) TB treatment make PN a significant concern for patients [4]. Our patient developed neuropathic symptoms prior to the initiation of treatment, necessitating an evaluation of various potential etiologies.

The progression of PN depends on the underlying etiology. Two studies published in 2020 demonstrated that early symptoms of TB start with progressive sensory disturbances, manifesting as tingling, numbness, burning pain, and dysesthesia in the fingers and toes, which may ascend to involve the arms and legs. In advanced stages, patients may develop complete loss of reflexes, the characteristic stocking-glove sensory loss, muscle wasting, and weakness [3,5].

Microbiological confirmation through sputum smear microscopy, culture, or PCR, supported by radiological findings, is essential for diagnosing TB. Histopathological confirmation via tissue biopsy is vital for diagnosing extrapulmonary TB [6]. Since TB can mimic or coexist with other neurological conditions, spinal involvement should always be ruled out once the disease is diagnosed. MRI provides superior visualization of bone loss, abscess formation, and spinal cord compression and remains the gold standard for identifying vertebral or neural involvement [7]. In our case, spinal TB was effectively ruled out by MRI, which showed no evidence of cord pathology or vertebral destruction. This exclusion supports a para-infectious immune-mediated mechanism.

TB rarely damages peripheral nerves directly, usually during extrapulmonary TB manifestations such as tuberculous cervical lymphadenitis in specific endemic regions. One documented case illustrated how the great auricular nerve became infected via nearby lymph node involvement, leading to neural invasion linked to inflammatory substances and mediators [8]. In our patient's case, the triple biopsy results showed no evidence of tuberculous involvement, and no lymphadenopathy was present to support nerve damage through direct spread.

Another proposed mechanism involves small-vessel vasculitis. In particular, leukocytoclastic vasculitis (LCV) represents a rare condition associated with TB infections. Patients with this condition exhibit both systemic symptoms and cutaneous manifestations. A study reported a patient with biopsy-proven cutaneous LCV and PN who received anti-TB treatment, resulting in symptom improvement [9]. The nerve biopsy results from our patient showed perivascular inflammation with no histological evidence of granulomatous changes. Additionally, the patient did not present with rash or purpura.

Systemic infections such as HIV and hepatitis C virus (HCV) are established causes of PN. The neurological symptoms of HIV-associated neuropathy include painful dysesthesia, burning sensations, and allodynia [10]. HCV can cause sensorimotor or small-fiber neuropathies through immune-mediated reactions, even in patients without cryoglobulinemia [11]. Our patient's neuropathic symptoms matched the typical presentation; however, both HIV and HCV serologies were negative, supporting the hypothesis that *Mycobacterium tuberculosis* potentially caused the para-infectious condition.

A recent investigation of patients with chronic idiopathic axonal polyneuropathy (CIAP), which is the same kind of axonal degeneration present in TB immune-mediated neuropathy, demonstrated a gradual yet quantifiable decline in nerve conduction amplitudes, approximately 4-6% annually, with an association with specific human leukocyte antigen (HLA) alleles [12]. This indicates the presence of an immune-mediated mechanism similar to that proposed in para-infectious neuropathies. Collectively, these findings imply that although axonal neuropathies may progress, prompt diagnosis and supportive intervention can preserve functionality and promote long-term recovery.

Our patient experienced substantial neurological improvement after initiating antituberculous therapy and returned to work for an extended period. This further supports the conclusion that the PN was para-infectious in origin and secondary to TB.

## Conclusions

This case represents a rare association between TB and PN unrelated to drug-induced neuropathy. It highlights the importance of considering *Mycobacterium tuberculosis* as a potential underlying cause of PN, particularly in patients presenting with neuropathic symptoms of unknown etiology after common causes have been excluded.
